# Evaluation of comparative effect between aluminum hydroxide gel and montanide (ISA 70) in potency and protection of locally prepared rabbit hemorrhagic disease virus 2 (RHDV2) vaccines in rabbits

**DOI:** 10.1186/s12917-024-04239-w

**Published:** 2024-09-11

**Authors:** Doha Abd Alrahman Ahmed, Yasmin Sadiek, Mostafa Saif Eldin, Ragab S. Ibrahim, Omar Amen, Samah El Sayed Ali Abodalal

**Affiliations:** 1 Avian and Rabbit Medicine Department, Faculty of Veterinary Medicine, Assiut University, Assiut, Egypt; 2 Colleague, Avian and Rabbit Medicine Department, Faculty of Veterinary Medicine, Assiut University, Assiut, Egypt; 3 Department of Poultry Viral Vaccines, Veterinary Serum and Vaccine Research Institute (VSVRI), Agricultural Research Center (ARC), Cairo, 11381 Egypt

**Keywords:** RHDV2, Vaccination, Montanide ISA70, Humoral immune response, Potency

## Abstract

**Background:**

Rabbit hemorrhagic disease (RHD) is an acute infectious disease that damages the rabbit industry by producing significant mortality rates in young and adult rabbits. RHD is better controlled by vaccination.

**Objective:**

The current study's goal was to prepare and evaluate the immuno-enhancing effect of montanide ISA70 and aluminum hydroxide (Al(OH)_3_) gel incorporated within the inactivated RHDV2 vaccine and assess the vaccine's protective efficacy against the homologous and heterologous local RHDV2 strains in rabbits.

**Methods:**

Inactivated RHDV vaccines were prepared using Montanide ISA70 oil or Al(OH)_3_ gel adjuvants and submitted to sterility, safety, and potency tests. 200 rabbits were equally divided into 4 groups: G1 (control), G2 (vaccinated with gel-incorporated vaccine), G3 (vaccinated with montanide-incorporated vaccine), and G4 (vaccinated with gel- and montanide-incorporated vaccines). Individual blood samples were collected from one week to six months from all groups. The vaccine's potency was measured by the HI test and protection percentage post challenge.

**Results:**

Data revealed slightly increasing HI titer means reaching the 1st peak at 4 weeks post-vaccination (7.33, 7.67, and 7.33 log2 in the 2nd, 3rd, and 4th groups, respectively), then slightly decreasing and peaked again, giving 9.33 log2 for the2^nd^ group at 3 months post-vaccination (MPV), 10.67 log2 for 3rd the group, and 10.33 log2 for the 4th group at 5 months post-vaccination. Titer gradually decreased but remained protective. The protection rate ranged from 80–100% and 80–90% for homologous and heterologous local RHDV2 vaccines, respectively, within 3 weeks and 6 months post-challenge. The montanide oil RHDV2 vaccine induced better protection than the aluminum gel RHDV2 vaccine.

**Conclusion:**

The results demonstrated evidence of cross-protection between RHDV2 strains. The oil emulsion vaccine induced higher and longer-lasting antibody titers than those obtained with the RHDV2 aluminum gel vaccine.

## Introduction

Rabbit viral hemorrhagic disease (RVHD) is an acute, highly contagious illness that causes hemorrhagic diathesis in all organs, particularly the liver and lungs. It is caused by a single-stranded positive-sense (ssRNA +) virus that is non-enveloped and belongs to the Caliciviridae family, specifically the genus Lagovirus. It is one of the factors that contribute to global economic losses in the rabbit breeding industry [1, 2].

A phylogenetic study reveals that RHDV strains are categorized into three types: the classical RHDV with genogroups G1-G5 RHDV1, the G6 variant strain (RHDV1), and the novel type RHDV2 [3, 4].

The RHDV2 was initially identified in 2010 in France [5] and subsequently found in other Lower Egypt governorates during 2018 and 2019[6, 7]. The mortality rate from RHDV2 infection varies and can reach between 5 and 70% in adult and nursing rabbits [5].

Initially, the Egyptian classical strain was utilized to prepare inactivated RHDV formalized vaccines [8]. Later, in 2006, RHDVa variant strains were discovered, and they started to substitute the classic RHDV strain in the vaccine's production in 2008 [9]. High mortality occurred in a number of rabbit flocks immunized with commercial vaccines made from either the classic or variant strains of RHDV (RHDV/RHDVa). This is because a novel RHDV strain called RHDV2 is antigenically different from the classical strain, and RHDVa and RHDV2 do not have cross-protection immunity [7, 10, 11].Subsequently, vaccinating rabbits with a vaccine containing both antigenic types (RHDVa and RHDV2) or a homologous strain to that observed during the outbreak was recommended according to the [18].

The duration of immunity and protection following vaccination depends on the type of adjuvant used; this duration is longer for oil emulsion vaccines [12].

Aluminum hydroxide gel is still in use in Egypt and is most frequently used as an adjuvant included in the inactivated RHDV vaccine [3, 8, 13–16].

Montanide incomplete seppic adjuvant (ISA) is an immunomodulator and an oil emulsifier used to create several oil-emulsion veterinary vaccines to enhance the immunological response [17]. Salman and Abodalal prepared inactivated RHDV oil emulsified (OE) vaccines were prepared using 4 different types of Montanide ISAs (SEPPIC, France) (ISA 70, ISA 206, ISA 71, and ISA 760). Montanide ISA 70 found to be the most suitable Montanide ISA followed by Montanide ISA 206 then Montanide ISA 760 and finally Montanide ISA 71 and could be used safely for active immunization of rabbits against this disease that threaten rabbit industry in Egypt [36].

The evaluation of protection percentage in a prior study was based upon homologous challenge against RHDV2 strains [22].

Therefore, the current study's aim was to prepare and evaluate the immuno-enhancing effects of ISA70 and aluminum hydroxide Al (OH)3 gel incorporated within the inactivated RHDV2 vaccine on rabbits and assess the homologous and heterologous vaccine's protective efficacy against local RHDV2 strains.

### Experimental animal

A total of 260 seronegative white New Zealand rabbits weighing between 1.5 kg and 6 weeks of age were provided by the Department of Poultry Viral Vaccines, Veterinary Serum and Vaccine Research Institute (VSVRI), Agricultural Research Center (ARC), Cairo, Egypt following rules for animal welfare. The HI test verified the rabbits' seronegative status for RHDV2 strains**.** These rabbits were needed in order to determine the lethal dosage 50 (LD50) and prepare and evaluate the vaccines. They were kept in disinfected metal cages in a well-ventilated and disinfected room, receiving commercial pellet rations and clean water ad libitum.

### Local RHDV2 strains

One locally isolated RHDV2 strain, designated Assuit.vac5 (accession number OQ925951), was identified through hemagglutination tests (HA) and RT-PCR in addition to sequence and phylogenetic analysis in accordance with the authors’ previous work and was used for vaccine preparation and the challenge of vaccinated rabbits. Mahala2019/VSVRI is a local Egyptian strain of RHDV2 (accession no. MK736667) with a titer of 10^6.7^ LD50/mL and a 2^12^ HA titer of HA units provided by, (VSVRI) for the challenge of vaccinated rabbits.

### RHDV2 propagation and determination of LD50

According to [18], fifty susceptible rabbits were used for propagation and determination of the LD50 for RHDV2 viruses.

### RHDV2 vaccine preparation

In the virology laboratory of (VSVRI), all procedures were carried out in accordance with the OIE Terrestrial Manual [18].Briefly, propagation of the virus was done in seronegative susceptible rabbits firstly, and subsequently formalin (Fluka Riedel–deHaen, Sigma, Germany Lot No. 52930) was added to the supernatant at a final concentration of 0.4% for 48 h at 37 °C. Throughout inactivation, the fluid was continuously mixed. In order to evaluate the inactivation of the virus, five rabbits were injected with an inactivated suspension, with two rabbits serving as controls. For two weeks, these rabbits were maintained under observation. If the infected rabbits showed no clinical signs of sickness or death, the inactivated suspension was considered ready for emulsification with a vaccine adjuvant. Two adjuvants were used for the preparation of two suspensions. The first suspension was adjuvanted with 2% aluminum hydroxide gel (occupying 20% of the vaccine volume), and the other suspension was adjuvanted with Montanide ISA 70 VG oil emulsion (occupying 70% of the preparation volume). Finally, the vaccination dosage was adjusted to HA units in 0.5 mL per rabbit and subcutaneously administered in two injections [18–20].

### Quality control assessment of the prepared vaccine

According to [18], the manufactured vaccines were examined for sterility, safety, and effectiveness. The vaccines were tested for sterility, or the absence of living fungi and bacteria. This was achieved through culturing on media like sabouraud dextrose and nutrient agar. Concerning the vaccine's safety, it was carried out by giving a double dose of the inactivated gel vaccine via the SC route to five seronegative rabbits and double dose of the inactivated oil vaccine via the SC route to five seronegative rabbits. After vaccination, these rabbits were observed for three weeks. Finally, the evaluation of the vaccine's effectiveness is based on the immunological response detected by means of the HI test and challenge test.

### Comparative assessment of the humoral immune response of aluminum hydroxide gel and montanide ISA 70 in a locally prepared RHDV2 vaccine

#### Adjuvants

Aluminum hydroxide (Al (OH)_3_) was supplied by CHEM TRADE-BERKELEY HEIGHTS, NEW JERSEY (Stock No. 203120070602). It was used according to the manufacturer's instructions.

Montanide ISA 70 VG was supplied by SEPPIC, PUTEAUX CEDEX, France (lot number T34651**) **and used according to the manufacturer's instructions.

#### Experimental design

Two hundred rabbits were equally divided into four groups. The first group (1st group) was the control unvaccinated group; the second group (2nd group) received 0.5 mL of inactivated adjuvanted aluminum hydroxide gel vaccination subcutaneously(SC); the third group (3rd group) received the same dose of the inactivated adjuvanted oil vaccine via SC vaccination; both the second and third groups will receive a booster dose after two weeks; and the fourth group received a 0.5 mL dose of an inactivated adjuvanted aluminium hydroxide gel vaccine and then received the same dose of an inactivated adjuvanted oil vaccine after two weeks. Each rabbit group was housed separately under well-hygienic measures and kept under daily observation till the end of the experiment.

#### Serum samples

The serum samples were collected from the vaccinated and non-vaccinated groups before vaccination (0 days), then “weekly to week 6”, then monthly, until the 6th month post-vaccination (MPV). Sera were separated and kept at -20 °C until used to evaluate the humoral immune response through an HI test.

### Assessment of humoral immune response among the vaccinated groups

#### Hemaggllutination (HA) test

According to [21], **a** two-fold serial dilution of the RHDV was incubated with an equivalent volume of 0.75% concentration washed human RBCs " type O" in a U-shaped-bottom micro-titer plate (Nunc, Roskilde, Denmark) at 4 °C to measure 8 HAU used in the HI test.

#### Hemagglutination inhibition (HI) test

The HI procedures were performed as previously reported [18]. In brief, a two-fold serial dilution of serum samples was carried out in 50 μl of PBS, and an equivalent volume of virus antigen was added that contained 8 HA units before incubation for 30 min at 37 °C. Human RBCs (type O) 0.75% were added (50 μl) and incubated for 1 h at 4 °C. The endpoint was the serum dilution revealing inhibition of hemagglutination, indicated as mean HI log2/ml titers.

#### The prepared vaccinations' challenge

At the 3rd WPV and at the 6th MPV, randomly chosen 20 rabbits from each group, either vaccinated (from 1st to 3rd) or unvaccinated (4th), were transported to experimental isolators, where they were subdivided into 2 groups. Ten rabbits from each group were challenged with intramuscular inoculation of 1 ml of a suspension with 10^3^ LD50/ml and 2^12^ HA units of a homologous locally isolated RHDV2 strain (Assuit.vac5), and the other ten rabbits were challenged with a heterologous local Egyptian strain of RHDV2 (Mahala2019/VSVRI) with a titer of 10^6.7^ LD50/ml and2^12^ HA units.

### Evaluation of protection percentage post challenge

For two weeks after the challenge, the survivals and deaths of the challenged rabbits were monitored daily. Serum samples were collected from survived rabbits at 1 and 2 weeks post challenge for the HI test [22].

$$\mathrm{Protection}\;(\%)=\frac{\mathrm{Number}\;\mathrm{of}\;\mathrm{survivors}\;}{\mathrm{Total}\;\mathrm{number}\;\mathrm{of}\;\mathrm{challenged}\;\mathrm{rabbits}}\mathrm X100$$

### Statistical analysis

For analyzing the HI test results, a one-way ANOVA was carried out, and Duncan’s multiple range test was used to detect significance between different treatments. A *P* value < 0.05 of the recorded data was considered statistically significant [23].The software used was GraphPad Prism version 9.0.0.

## Results

### Assessment of virus inactivation and titration of RHDV-2 strains

Each of the rabbits injected with the formalin-inactivated virus was still alive and did not exhibit any clinical signs. The titer of the homologous locally isolated RHDV2 strain (Assuit.vac5) was 10^3^ LD50/ml and2^12^ HA.

### Sterility and safety of locally prepared inactivated RHDV2 vaccines

Based on the results of the sterility test, there was no bacterial or fungal contamination of the homologous vaccines that were manufactured. According to the results of safety testing, the prepared vaccines were safe. After receiving a double field dose of the vaccine, the inoculated seronegative susceptible rabbits exhibited no clinical signs for three weeks post-vaccination.

### Potency

The immune response of locally prepared RHDV-2 vaccines was confirmed by the HI test. Serum samples were collected from each group within (1WPV to 6MPV). Prior to vaccination, the HI test verified that none of the groups had a RHDV2 antibody titer. Each immunization group exhibited antibody titers that were protective.

The mean antibody titers against RHDV2 for 3rd group and 4th group were slightly higher than for 2nd group (WPV), which were 4, 4.67, and 4.33 log2 for the 2nd,3rd and 4th groups, respectively, after one week post-vaccination.

The mean HI titers were slightly increasing, reaching a peak of 7.33, 7.67, and 7.33 log2 for the 1st group, 2nd group, and 3rd group, respectively at 4WPV, then slightly decreased and peaked again, giving 9.33 log2 for the 2nd group at 3 months post-vaccination (MPV), 10.67 log2 for the 3rd group, and 10.33 log2 for the 4th group at 5MPV. Titer gradually decreased but remained protective.

At the end of the experimentation (6 months), mean HI antibody titers ranged from as low as 6.33 log2 in the 2nd group to as high as 10.33 log2 and 10 log2 for the 3rd and 4th groups, respectively.

The results showed that there were significant differences (*p* < 0.05) between the 2nd, 3rd, and 4th groups from 1WPV to 6MPV, but only slightly significant differences between the 3rd and 4th groups.

**Fig. 1:**
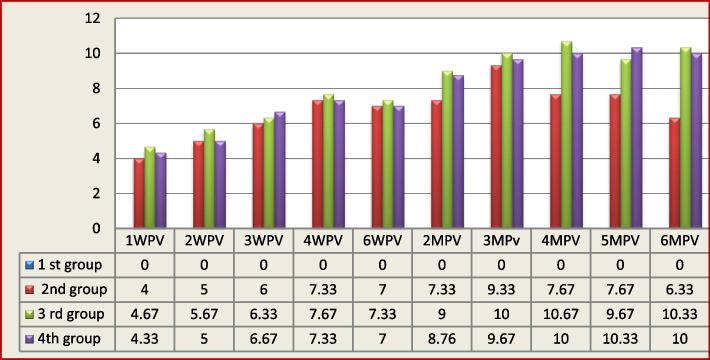
Means of HI antibody titers (log2) in the sera of vaccinated and unvaccinated rabbits

These findings demonstrated that, in comparison with the combination of Gel + Oil and aluminum hydroxide gel adjuvanted vaccine, the oil adjuvanted vaccination produced an earlier and higher humoral immunity, as shown in Fig. 1.

### Protection

The protection rate is nearly identical, ranging from 80–100% and 80–90% for homologous and heterologous local RHDV2 vaccines, respectively, within 3WPC and 6MPC. This demonstrates evidence of cross-protection between RHDV2 strains.

The protection rate ranged from 80 to 90% and 90–100% in rabbits vaccinated with aluminum hydroxide gel and oil emulsified vaccine, respectively within 3WPC and 6MPC.

There were very significant differences between the 2nd group (vaccinated with aluminum gel), the 3rd group (vaccinated with montanide oil), and the 4th group (combination (gel + oil)) within 3WPC and 6MPC, but only slightly significant differences between the 3rd and 4th groups. So, challenged rabbits in the group vaccinated with the Montanide Oil RHDV2 vaccine exhibited more protection (%) than the Aluminum Hydroxide Gel RHDV2 vaccine, as shown in Tables 1 and 2. In contrast, in the unvaccinated challenged group (the control positive group), the mortality rates were 100% within 3 days of the challenge, with typical signs and lesions for RHD.

**Table 1:** The mean of HI antibody titers (Log2 ± SE) in the sera of challenged rabbits

Time of challenge	Time after challenge	Challenged strains	2nd group	3rd group	4th group
**3rd week**	1st week	Assuit	4.667 ± 0.67^b^	8.667 ± 0.33^a^	9.333 ± 0.33^aa^
		Mahala	4.333 ± 0.33^b^	7.667 ± 0.33^a^	8.333 ± 0.33^a^
	2nd week	Assuit	6.667 ± 0.33^b^	10.33 ± 0.33^aa^	9.667 ± 0.33^ab^
		Mahala	7.333 ± 0.33^b^	9.667 ± 0.33^aa^	9 ± 0.00^ab^
**6th month**	1st week	Assuit	4.667 ± 0.33^b^	8.667 ± 0.33^a^	8.667 ± 0.33^a^
		Mahala	4.333 ± 0.33^b^	8 ± 0.57^aa^	6.667 ± 0.33^ab^
	2nd week	Assuit	5.333 ± 0.33^b^	9.667 ± 0.33^aa^	7 ± 0.57^ab^
		Mahala	6 ± 0.57^b^	9.667 ± 0.33^aa^	7 ± 0.57^ab^

2nd group: vaccinated with aluminum hydroxide gel, 3rd group: vaccinated with montanide oil, 4th group: combination (gel + oil), *p* value < .005, a,b,c: significant difference between groups, Data are expressed as mean ± SE

**Table 2:** Protection percentage of rabbits immunized with locally produced RHDV2 vaccines after challenge

	protection%(n/t)		protection%(n/t)	
	**3rd week post challenge**		**6th month post challenge**	
	**Assuit strain**	**Mahala strain**	**Assuit strain**	**Mahala strain**
**Control**	0(0/10)	0%(0/10)	0%(0/10)	0%(0/10)
**Gel**	90%(9/10)	80%(8/10)	80%(8/10)	80%(8/10)
**Oil**	100%(10/10)	90%(9//10)	100%(10/10)	90%(9/10)
**Gel + Oi**	100%(10/10)	90%(9/10)	100%(1010)	90%(9/10)

(n/t) refer to No of survival rabbits/No of challenged rabbits

## Discussion

Vaccination is considered the most effective approach for reducing morbidity and mortality associated with epidemics [3]. Despite control measures, the virus has still managed to spread widely within a short period of time after the emerging of new RHDV2 strains in Egypt during 2018 [7].

The broad use of inactivated RHDV vaccine in Egypt, either locally produced (with an Al (OH) 3 gel adjuvant) or imported from Spain (with an oil adjuvant called CUNIPRAVAC-RHD), is essential to the control of RVHD [3].

Updating the current situation is very essential for resolving this problem and establishing a new plan to control this disease. So the current study's goal was to prepare and evaluate the immuno-enhancing effects of ISA70 and aluminum hydroxide (Al(OH)3) gel incorporated within inactivated RHDV vaccines on vaccinated rabbits and assess the homologous and heterologous vaccines's protective efficacy against local RHDV2 strains.

According to [18], evaluation of inactivated vaccine potency is mainly based on the immune response of locally prepared RHDV-2 vaccines, which was confirmed by HI testing. Individual blood samples were collected from one week to six months from all groups.

In our studies, all immunization groups showed protective antibody titers (above 2^4^), but the non-immunized group (control) showed no noticeable RHDV2 immune responses. This is in accordance with Salman [3], who reported that the protective titer of an antibody is 2^4^, below which the titer is considered non-protective.

The obtained results revealed that the mean antibody titers against RHDV2 for 3rd group and 4th group were slightly higher than for 2nd group, which were 4, 4.67, and 4.33 log2 for the 2nd, 3rd, and 4th groups, respectively, after one week post-vaccination (WPV). The specific antibody titer for RHDV2 was lower than the value obtained by [24], who said that the mean titers at 1st WPV for locally prepared RHDV2 vaccine also induce a protective and rapid immune response in the vaccinated rabbits. The same results were recorded by [22], who recorded the titer for the RHDV2 vaccine. Additionally, [25], who demonstrated mean titers of RHDV antibodies for RHDV2 at 1st WPV.

The mean HI titers were slightly increasing, reaching a peak of 7.33, 7.67, and 7.33 log2 for the 2nd group, 3rd group, and 4th group, respectively at 4WPV, then slightly decreased and peaked again, giving 9.33 log2 for the 2nd group at the 3 month post-vaccination (MPV), 10.67 log2 for the 3rd group, and 10.33 log2 for the 4th group at 5MPV. Titer gradually decreased but remained protective. This pattern of fluctuation in rising and decreasing HI-antibody titers was in full agreement with [20, 34, 35], who indicated that the oil emulsion vaccine caused an antibody titer to develop in a zigzag pattern. This finding aligned with [9, 33], who demonstrated that the RHDV2 antibody levels increased gradually, then decreased, and HI antibody titers reached 2^8^ at the 3rd WPV. Additionally, [24] who reported that the mean antibody titers for RHDV2 gradually and significantly rose, reaching 2^10.6^ for locally, prepared RHDV2 vaccine at 3rd WPV.

Our results were nearly matched with others in the mean of HI titers, but there were differences in the duration of reaching peak HI titers. This could indicate that there were many factors affecting rabbit breeds, including environmental factors like stress, seasons, medications, and the usage of different types of adjuvants.

The results showed that there were significant differences (*p* < 0.05) between the 2nd, 3rd, and 4th groups from 1WPV to 6MPV, but only slightly significant differences between the 3rd and 4th groups. These findings demonstrated that, in comparison with the combination of Gel + Oil and aluminum hydroxide gel adjuvanted vaccines, the oil adjuvanted vaccination produced an earlier and higher humoral immunity. These outcomes corroborated [27], who said that the quantity of eluted antigen from aluminum salts may be considerably reduced by the adsorption of antigen onto adjuvants containing aluminum, resulting in a poor antibody response and an increasing antibody titer following vaccination with an oil-emulsified vaccine due to its high homogenicity and low viscosity. These results were consistent with those of [22], who observed that from the first WPV until the end of the experiment, vaccination with montanide ISA oil showed an earlier and better immune response due to the slow release of the oil adjuvant vaccine antigen. These outcomes matched those of [32], who reported that both humoral and cellular reactions are induced by the oil emulsion vaccination. Similar findings achieved by [31], who concluded that the VP60 protein of RHDV is capable of inducing potential humoral and cell-mediated immune response immunization. In addition [30], showed that the advantages of emulsions include enhanced antibody production through prolonged release of antigen.

At end of experimentation (6 months), vaccinated rabbits had a mean HI antibody titers ranging from as low as 6.33 log2 in 2nd group to as high as 10.33 log2 and 10 log2 for 3rd group and 4th group, respectively. The same results recorded by [25] who said that the oil-emulsion vaccine provided a slow but long-term (1 year) immune response, whereas the gel-based vaccine (water-based) provided a fast but shorter-term (6 months) immune response.

The prepared vaccines were potent and provided 80–100% protection. This was in accordance [18], which showed that the vaccination must provide at least 90% protection.

In our observation.There were very significant differences between the 2nd group (vaccinated with aluminum gel), the 3rd group (vaccinated with montanide oil), and the 4th group (combination (gel + oil)) within 3WPC and 6MPC, but only slightly significant differences between the 3rd and 4th groups. So, challenged rabbits in the group vaccinated with the Montanide Oil RHDV2 vaccine exhibited more protection (%) than the Aluminum Hydroxide Gel RHDV2 vaccine,

The protection rate ranged from 80 to 90% and 90–100% in rabbits vaccinated with aluminum hydroxide gel and oil emulsified vaccine, respectively, within 3WPC and 6MPC. These findings were consistent with those of [25, 28, 29], who observed 100% protection in rabbits challenged with a virulent RHDV2 strain 7 days after vaccination. The same results were also recorded by [22], whoindicated that the oil-emulsion vaccine exhibited 100% protection and induced a high antibody titer due to its adequate antigen concentration. Furthermore, these findings aligned with [27], who reported that protective immunity against RHDV-2 took more than a week to develop because the aluminum hydroxide gel relies on the depot effect to trigger the immune response.

The protection rate is nearly identical, ranging from 80%–100% and 80–90% for homologous and heterologous local RHDV2 vaccines, respectively, within 3WPC and 6MPC. This demonstrates evidence of cross-protection between RHDV2 strains, but there is no cross-protection between RHDV1 and RHDV2 [24, 26]. So, it is recommended to vaccinate rabbits with a vaccine containing the same strain that was detected during the outbreak or a bivalent vaccine containing both antigenic types (RHDV1 and RHDV2) to control both RHD virus outbreaks in Egypt and minimize economic losses.

In our study, the unvaccinated challenge group (the control positive group) had 100% mortality rates within 3 days of challenge with typical signs and lesions for RHD. This result is in accordance with [25].

## Conclusion

Vaccination is the best way to minimize the morbidity and mortality associated with epidemics. The results demonstrated that the locally prepared vaccines achieved a high degree of protection. Evidence of cross-protection between RHDV2 strains is demonstrated by the findings, but there is no cross-protection between RHDV1 and RHDV2. To prevent both RHD virus outbreaks in Egypt and reduce economic losses, it is advised to vaccinate rabbits with a vaccine containing the strain that was discovered during the outbreak or a bivalent vaccine comprising both antigenic types (RHDV1 and RHDV2). Compared to the RHDV aluminum hydroxide gel vaccine, the oil emulsion vaccine produced higher and longer-lasting antibody titers; therefore, it was suggested that montanide ISA 70 can be used as an adjuvant in the vaccine formulation instead of aluminum hydroxide gel.

## Data Availability

All data generated or analyzed during this study are available from the corresponding author on reasonable request
